# Validation of Chinese version of the 5-item WHO well-being index in type 2 diabetes mellitus patients

**DOI:** 10.1186/s12888-023-05381-9

**Published:** 2023-11-29

**Authors:** Jianhua Du, Yinan Jiang, Cathy Lloyd, Norman Sartorius, Jie Ren, Weigang Zhao, Jing Wei, Xia Hong

**Affiliations:** 1 Department of Psychological Medicine, Peking Union Medical College Hospital, Chinese Academy of Medical Sciences & Peking Union Medical College, Beijing, People’s Republic of China; 2 Faculty of Wellbeing, Education and Language Studies, The Open University, Milton Keynes, UK; 3 Association for the Improvement of Mental Health Programmes (AMH), Geneva, Switzerland; 4 Department of Psychiatry, Beijing Xicheng District Pingan Hospital, Beijing, People’s Republic of China; 5 Department of Endocrinology, Peking Union Medical College Hospital, Chinese Academy of Medical Sciences & Peking Union Medical College, Beijing, People’s Republic of China

**Keywords:** WHO-5, Depression, Type 2 diabetes, Validity, Reliability

## Abstract

**Background:**

For better disease management and improved prognosis, early identification of co-morbid depression in diabetic patients is warranted. the WHO-5 well-being index (WHO-5) has been used to screen for depression in diabetic patients, and its Chinese version (WHO-5-C) has been validated. However, its psychometric properties remain to be further validated in the type 2 diabetes patient population. The aim of our study was to examine the reliability and validity of the WHO-5-C in patients with type 2 diabetes mellitus.

**Methods:**

The cross-sectional study was conducted on 200 patients from July 2014 to March 2015. All patients should complete the WHO-5-C, the Patient Health Questionnaire-9 (PHQ-9), the 20-item Problem Areas in Diabetes Scale (PAID-20), the Mini International Neuropsychiatric Interview (M.I.N.I), and Hamilton Rating Scale for Depression (HAM-D). Internal consistency of WHO-5 was revealed by Cronbach’s alpha, and constructive validity by confirmatory factor analysis (CFA). Relationship with PHQ-9, HAM-D, and PAID-20 was examined for concurrent validity, and ROC analysis was performed for criterion validity.

**Results:**

The WHO-5-C presented satisfactory reliability (Cronbach’s alpha = 0.88). CFA confirmed the unidimensional factor structure of WHO-5-C. The WHO-5-C had significant negative correlation with HAM-D (*r* = -0.610), PHQ-9 (*r* = -0.694) and PAID-20 (*r* = -0.466), confirming good concurrent validity. Using M.I.N.I as the gold standard, the cut-off value of WHO-5-C was 42, with a sensitivity of 0.83 and specificity of 0.75.

**Conclusion:**

The WHO-5-C holds satisfactory reliability and validity that is suitable for depression screening in type 2 diabetes patients as a short and convenient instrument.

## Background

Diabetes is recognized as one of the most challenging chronic diseases worldwide, with the global prevalence rising from 9% in 2019 to 10.5% in 2021 according to the International Diabetes Federation [1]. People with diabetes not only face life-threatening symptoms and disabling complications but may also suffer from psychosocial problems. According to the International Prevalence and Treatment of Diabetes and Depression Study (INTERPRET-DD), which spans 14 countries, the prevalence of depression in patients with diabetes reached 10.6%, with 17% of patients experiencing moderate to severe depressive symptoms [2]. In China, the combined prevalence of depression in patients with type 2 diabetes was 25.9%, ranging from 0.8 to 52.6%, according to different depression screening tools [3].

Numerous shreds of evidence have proved that depression may adversely influence glycemic control, comorbidity, and even mortality of diabetes [4–10]. Depression may also interrupt patients’ behavior and well-being, including diabetes self-care, diet control, physical exercise, and self-efficacy [11–15]. On the contrary, managing the psychosocial problems in diabetes mellitus can promote glucose control, dietary behavior, and life quality [16]. Various aspects of psychosocial well-being, such as self-efficacy, positive affect, optimism and resilience have indicated positive correlations with superior diabetes management and better outcomes [17–19]. Under these circumstances, screening for depression and identifying well-being status are of great significance for diabetes mellitus patients. Thus, proper screening instruments are essential to help both endocrinologists and psychiatrists identify the depressive disorders in these patients to provide timely intervention.

Several indices have been developed to evaluate psychological well-being, among which the 5-Item WHO Well-Being Index (WHO-5) has displayed satisfactory sensitivity and validity measurement of subjective well-being [20, 21]. All the item content of WHO-5 focused on positive well-being, such as “cheerful and in good spirits”, and “calm and relaxed”, which is rather different from other depression screening instruments. Thus, WHO-5 can be also used as a reliable screening tool for depression, as confirmed in various studies related to diabetes with good psychometric properties [20, 22]. Meanwhile, the psychometric properties of the Chinese Version of WHO-5 (WHO-5-C) have also been validated in a sample of 1414 Chinese university students [23]. However, it has not been implicated in a medical setting concerning diabetes. Given that Chinese are usually ashamed to express and admit bad mental feelings, WHO-5 is more acceptable culturally. On the other side, the WHO-5 is brief and efficient for assessment with only 5 items, thus measured conveniently in bustling Chinese clinics. The acceptance and effectiveness of the screening instrument should not be based merely on its psychometric properties, but take culture, language, and literacy into consideration.

Therefore, the purpose of this study was to explore the psychometric properties of the WHO-5-C in screening for depression in patients with type 2 diabetes.

## Methods

### Participants

The cross-sectional study was conducted to evaluate the reliability and validity of the Chinese Version of the 5-item WHO Well-Being Index in type 2 diabetes patients. All participants were recruited from the Department of Endocrinology in Peking Union Medical College Hospital from July 2014 to March 2015.

Inclusion criteria: 1) aged between 18 to 65 years old; 2) diagnosed with type 2 diabetes for at least 1 year; 3) outpatients; 4) could sign the informed consent independently.

Exclusion criteria: 1) diagnosed as type 2 diabetes within 1 year; 2) illiterate, cognitively impaired, or patients in serious medical condition (e.g., delusional, delirium, or acute suicidal tendency) that could not complete the survey; 3) hospitalized patients; 4) involved in other interventional studies within 3 months; 5) pregnant or within the six months of lactation.

Ethical approval was obtained from the institutional review board of Peking Union Medical College Hospital (registration number S-713) as part of the International Diabetes and Depression Comorbidity and Treatment research, and written informed consent was obtained from all the participants.

### Procedure

Before completing the questionnaires, sociodemographic information such as sex, age, education level, marriage state, living site, and income level was collected from patients. Questionnaires were then provided to patients to finish, including the WHO-5-C, the 9-item Patient Health Questionnaire (PHQ-9), and the 20-item Problem Areas in Diabetes (PAID-20). After finishing the questionnaires, patients were then assessed by a research assistant who was unaware of the patients’ WHO-5-C scores and was trained in the use of the Mini International Neuropsychiatric Interview (M.I.N.I.), Hamilton Rating Scale for Depression (HAM-D).

### Measures

The WHO-5 consists of five items with a 6-point Likert-type scale ranging from 0 (at no time) to 5 (all of the time) that measure well-being. The raw score ranging from 0 to 25 was then multiplied by 4 to give the final score. A higher score indicates a higher level of well-being [20]. The WHO-5-C experienced translation and back-translation procedures by two individual translators and was further convinced in two pilot studies, reporting no difficulty in understanding the questions [24]. Internal consistency was confirmed in several studies [23].

The M.I.N.I. is a structured clinical diagnostic interview schedule standardized for the Diagnostic and Statistical Manual of Mental Disorders, 4th Edition Axis-I Disorders [25]. It can be carried out reliably by properly trained interviewers. The depression modules of the schedule were used in the study.

The HAM-D is a mature tool for depression severity evaluation and prognosis prediction. The Chinese version of the HAM-D has acceptable inter-item consistency (Cronbach’s α = 0.714) and concurrent validity, holding a negative correlation with the Global Assessment Scale (Spearman’s correlation coefficient = -0.487, *p* < 0.001) [26].

The Chinese version of PAID-20 measures emotional problems in diabetes patients by a 20-item self-report scale. A positive relationship with Hb_A1C_ was revealed was reported (Spearman’s correlation coefficient = 0.15, *p* < 0.05), indicating that levels of diabetes-related distress were related to poor glycemic control [27].

PHQ-9 has been developed as a valid screening tool for depression, broadly utilized in primary care settings. The Chinese version of PHQ-9 has been validated with Cronbach’s α ranging from 0.765 to 0.983, pooled sensitivity of 0.88 (95% CI 0.80–0.87), and pooled specificity of 0.87 (95%CI 0.83–0.91), proving its significance for clinical use [28].

### Statistical analysis

Statistical Package Social Sciences (SPSS) 25.0 statistical software and Mplus 8.8 and AMOS 28 were employed to perform statistical analyses.

SPSS was used to establish internal consistency reliability by Cronbach’s α value. Cronbach’s α values of 0.70 were regarded as acceptable and 0.90 as excellent [29]. Mplus and AMOS were used to run a confirmatory factor analysis [estimation method = diagonal weighted least square] to test the factorial structure of the WHO-5-C in the Chinese diabetes samples. The Comparative Fit Index (CFI), Tucker-Lewis Index (TLI), and the root mean square error of approximation (RMSEA) were used to test global model fits. A good fit for continuous data is indicated for Comparative Fit Index > 0.95 [30], Tucker-Lewis Index > 0.95 [31], and root mean square error of approximation < 0.08 [32].

The concurrent validity of the WHO-5-C was analyzed by estimating the relationship of WHO-5-C with HAM-D, PHQ-9, PAID-20, and clinical indicators. The criterion validity of the WHO-5-C was tested using the relevant sections of the M.I.N.I. DSM-IV depression and PHQ-9. The receiver operating characteristic (ROC) curve was used to determine the level of accuracy with which the WHO-5-C can predict depression diagnosis.

## Results

### Characteristics of the patients with type 2 diabetes

A total of 200 patients were included. Among the participants, the mean age was 53.48 ± 9.76, with a range between 28.0 to 65.0, and 52.5% were male patients. Other demographic characteristics of the participants were presented in Table 1.

**Table 1:** demographic characteristics of the participants

		**MEAN ± SD (range)**
**Age**	years	53.48 ± 9.76 (28.0–65.0)
**HbA**_**1c**_	%	7.47 ± 1.90 (2.7–18.7)
		**N (n%)**
**Gender**		
Female		94 (47.0)
Male		106 (53.0)
**Highest level of education attained**		
Illiteracy		3 (1.5)
Primary school		11 (5.5)
Secondary school		85 (42.5)
Higher education		101 (50.5)
**Marriage**		
Married/co-habited		181 (90.5)
Single		7 (3.5)
Widowed		6 (3.0)
Divorced		6 (3.0)
**Family income**		
Unfixed		28 (14.0)
Fixed		172 (86.0)
**Location of residence**		
Country		29 (14.5)
Urban		171 (85.5)

*HbA*_*1c*_ glycated hemoglobin A1c, *SD* standard deviation

### Reliability of the WHO-5-C

The mean total WHO-5-C score of our sample was 60.72 (SD = 25.16) with a range of 0 to 100,

The mean scores for each WHO-5-C item in this study are shown in Table 2. The corrected item-total correlations for the WHO-5-C ranged from 0.63 to 0.75. The Cronbach’s α coefficient for the total scale was equal to 0.88, above the acceptable range.

**Table 2:** Chinese Version of the WHO-5-C item level values

Item	Mean (SD)	Skewness (SE)	Kurtosis (SE)	Cor_iT_	Cron. α_id_
1	12.66 (5.84)	-0.71 (0.17)	-0.56 (0.34)	0.71	.85
2	12.70 (5.69)	-0.80 (0.17)	-0.31 (0.34)	0.69	.85
3	11.62 (6.33)	-0.43 (0.17)	-0.86 (0.34)	0.74	.84
4	12.28 (6.19)	-0.56 (0.17)	-0.73 (0.34)	0.63	.87
5	11.84 (6.49)	-0.41 (0.17)	-1.10 (0.34)	0.75	.84
Total	60.72 (25.16)	-0.51 (0.17)	-0.63 (0.34)		

WHO-5-C = Chinese version of the 5-item WHO Well-Being Index; *Cor*_*iT*_ corrected item total correlation, *Cron. α*_*id*_ Cronbach α if item deleted, *SD* standard deviation

### Validity of the WHO-5-C

#### Construct validity

**Fig. 1:**
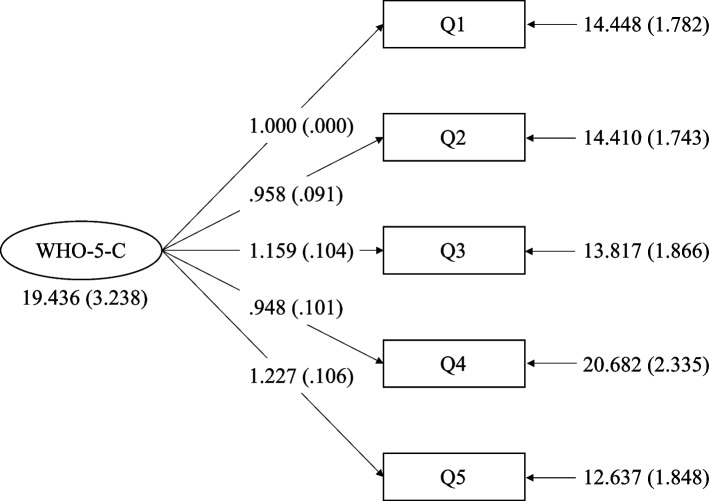
Factor structure of the WHO-5-C. WHO-5-C: Chinese version of the 5-item WHO Well-Being Index

The Kaiser–Meyer–Olkin measure of sampling adequacy (KMO) was 0.869, indicating sample adequacy; Bartlett’s test of sphericity was 472.911 (df = 10, *p* < 0.001), suggesting that factor analysis was justified in the sample. Since the one-factor model has been confirmed in WHO-5-C by other researchers, a CFA with weighted least square estimation was conducted to examine the factorial validity of the WHO-5-C in diabetes mellitus patients. The analysis showed that the factor loading of each item in the model was above 0.4, as depicted in Fig. 1. Fitting the one-factor WHO-5-C model to the present sample yielded a good fit to the data with a ratio of χ 2 to df = 1.3, RMSEA = 0.038 (by AMOS) or 0.039 (by Mplus), TLI = 0.994, and CFI = 0.997 (see Table 3), which were considered a good fit.

**Table 3:** Fit indices for unidimensional CFA model of the WHO-5-C in diabetes mellitus sample

Fit indices	AMOS	Mplus	Cutoff Values
Ratio of χ 2 to df	1.3	1.3	≤ 2 or 3
Root Mean Square Error of Approximation	0.038	0.039	< 0.08
Tucker–Lewis fit Index	0.994	0.994	≥ 0.95
Comparative fit Index	0.997	0.997	≥ 0.95

*WHO-5-C* Chinese version of the 5-item WHO Well-Being Index

### Concurrent Validity

Using Pearson’s correlation coefficient to determine the concurrent validity, the score of each item and the total score of the WHO-5-C are all negatively correlated with the total score of HAM-D, PHQ-9, and PAID-20. The correlation coefficients were listed in Table 4.

**Table 4:** Correlation coefficients between WHO-5-C items and HAMD, PHQ-9 and PAID-20

	**WHO-1**	**WHO-2**	**WHO-3**	**WHO-4**	**WHO-5**	**WHO-total**
HAM-D	-.509^**^	-.400^**^	-.531^**^	-.554^**^	-.515^**^	-.610^**^
PHQ-9	-.518^**^	-.499^**^	-.566^**^	-.569^**^	-.575^**^	-.694^**^
PAID-20	-.376^**^	-.416^**^	-.387^**^	-.435^**^	-.336^**^	-.466^**^

WHO-1: First question of WHO-5-C = 5-item WHO Well-Being Index score

WHO-2: Second question of WHO-5-C = 5-item WHO Well-Being Index score

WHO-3: Third question of WHO-5-C = 5-item WHO Well-Being Index score

WHO-4: Fourth question of WHO-5-C = 5-item WHO Well-Being Index score

WHO-5: Fifth question of WHO-5-C = 5-item WHO Well-Being Index score

WHO-total: WHO-5-C = 5-item WHO Well-Being Index total score

HAM-D: Hamilton Rating Scale for Depression

PHQ-9: the 9-item patient health questionnaire

PAID-20: the 20-item problem areas in diabetes

^**^p < 0.001

We also explored the association between WHO-5-C total score and glycosylated hemoglobin, total cholesterol, low-density lipoprotein, high-density lipoprotein, triglyceride, and body weight. However, no significant correlation was identified as shown in Table 5.

**Table 5:** Mean, SD and correlation matrix of study variables

Variable	WHO-1	WHO-2	WHO-3	WHO-4	WHO-5	WHO-total	BMI	HbA_1c_	LDL-C	HDL-C	TC	TG
WHO-1					-							
WHO-2	.611**					-						
WHO-3	.592**	.569**										
WHO-4	.520**	.505**	.564**									
WHO-5	.619**	.617**	.701**	.545**								
WHO-total	.805**	.789**	.831**	-.759**	-.862							
BMI	.057	-.024	.002	.058	-.035	.014						-
HbA_1c_	.021	-.065	-.063	-.093	-.048	-.061	.004					
LDL-C	-.074	.009	.037	-.046	.023	-.015	.013	.061				
HDL-C	-.062	.143	.028	.029	.013	.046	-.225**	-.072	.177*			
TC	-.102	-.056	.018	-.061	.041	-.034	-.055	.066	.887**	.286**		
TG	-.029	-.180	-.107	-.969	-.021	-.098	.036	.125	-.039	-.310**	.194*	
Mean	12.66	12.70	11.62	12.28	11.84	60.72	25.65	7,47	2.66	1.15	4.49	1.71
SD	5.84	5.69	6.33	6.19	6.49	25.16	3.58	1.90	.71	.27	.89	1.08
N	200	200	200	200	200	200	200	172	149	147	150	150

WHO-1: First question of WHO-5-C = 5-item WHO Well-Being Index score

WHO-2: Second question of WHO-5-C = 5-item WHO Well-Being Index score

WHO-3: Third question of WHO-5-C = 5-item WHO Well-Being Index score

WHO-4: Fourth question of WHO-5-C = 5-item WHO Well-Being Index score

WHO-5: Fifth question of WHO-5-C = 5-item WHO Well-Being Index score

WHO-total: WHO-5-C = 5-item WHO Well-Being Index total score

*BMI* body mass index

*HbA*_*1c*_ glycated hemoglobin A1c

*LDL-C* Low-Density Lipoprotein Cholesterol

*HDL-C* High-Density Lipoprotein Cholesterol

*TG* Triglycerides

*TC* total Cholesterol

^*^p < 0.5

^**^p < 0.01

### Criterion Validity

The M.I.N.I. was used as a gold standard in determining the screening properties of WHO-5-C. Meanwhile, the criterion validity with PHQ-9 as a diagnostic standard was also examined.

According to the M.I.N.I., 28 patients (14.0%) met the diagnosis of major depression. It was illustrated from the ROC curve that the best diagnostic performance of WHO-5-C was achieved at a cut-off score of 42 (sensitivity = 0.83, specificity = 0.75, and Youden index = 0.58) in identifying patients with depressive disorders (Fig. 1). The area under the curve (AUC) in this study was 0.88 (SD = 0.03, 95% CI 0.81 to 0.94). Middle-range sensitivity and specificity were demonstrated in Table 6.

**Table 6:** sensitivity and specificity for the WHO-5-C within the Middle Range with MINI as diagnostic standard

Cutoff	Sensitivity	1-Specificity	Youden Index
18	0.977	0.607	0.37
22	0.953	0.536	0.417
26	0.942	0.5	0.442
30	0.913	0.429	0.484
34	0.884	0.321	0.563
38	0.878	0.321	0.557
42	0.831	0.25	0.581
46	0.814	0.25	0.564
50	0.785	0.214	0.571

*WHO-5-C* Chinese Version of 5-item WHO Well-Being Index

**Fig. 2:**
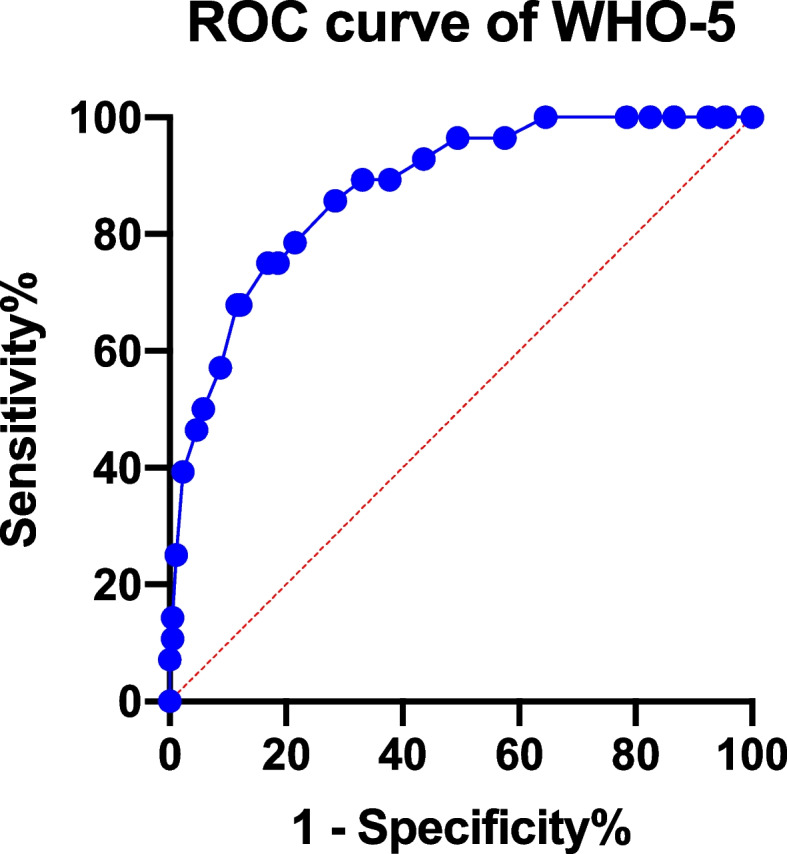
Diagnostic performance of WHO-5-C. WHO-5-C = Chinese version of 5-item WHO Well-Being Index

Using PHQ-9 ≥ 10 as a diagnostic standard, 37 patients (18.5%) met the criteria, and the cut-off score of WHO-5-C reaching the best diagnostic performance was 54 (sensitivity = 0.87, specificity = 0.75, and Youden index = 0.61). As was shown in Fig. 2, the AUC was 0.860 (SD = 0.04, 95% CI 0.79 to 0.93).

## Discussion

Our findings suggest good reliability and validity for the Chinese Version of WHO-5 and support its utility as a suitable screening instrument for likely depression among Chinese patients with type 2 diabetes. The WHO-5-C presents good internal consistency, with Cronbach’s alpha values of 0.85, similar to the values reported in other countries [33–35] and other medical settings in China [36, 37]. Construct validation was confirmed by CFA in our study. The results show WHO-5-C fits the unidimensional factor model well among diabetic patients, consistent with that of the original or other versions of WHO-5-C [20].

We also determined the concurrent validity of the WHO-5-C in relation to the HAM-D and PHQ-9, both obtaining significant negative correlations (*r* = -0.610 and -0.694, respectively), which indicates the comparative role of WHO-5-C as a screening instrument for depression in diabetes patients. This result is consistent with that of the original or other language versions of WHO-5 in diabetic patients [29, 38–42]. The correlation coefficient of WHO-5-C with PAID is relatively low (*r* = -0.466), but also significant (*p* < 0.001). PAID evaluates diabetes-related distress specifically, an adverse psychological burden mainly on account of rigorous diabetes management and worries about complications. WHO-5 assesses depression, a persistent low mood, and a lack of interest and motivation. The psychological characteristics reflected in the two scales partly overlap, but are different, which may contribute to the above lower coefficient value. We failed to reveal a significant correlation between the WHO-5-C score and other laboratory indicators, such as glycosylated hemoglobin. However, Papanas and Prinz’s studies both confirmed the significant association between WHO-5 and glycemic control [43, 44]. Our previous findings also suggest that depression and diabetes-related distress interact to affect glycemic control. Another cross-sectional study even indicated that the score of WHO-5 was reversely correlated with the number of diabetes complications [45]. We assumed that the reason for this discrepancy may lie in that how the psychological state affects the physical indicators is still unclear. Personality traits, coping styles, and resource support may all play a role in it. A further longitudinal study is required to clarify the predictive validity of WHO-5 for glycemic control and diabetes complications.

The ROC analysis indicated that the Chinese version of WHO-5-C has sufficient power to screen major depression patients from all diabetes mellitus patients. The AUC was close to 1, indicating satisfactory accuracy for diabetes screening. We discovered that at a cut-off point of < 42, the WHO-5-C had the best sensitivity (83.1%) and specificity (75.0%). However, the cut-off value is different from the original version of WHO-5-C, which is < 52 [20]. Most of the studies about other language versions of WHO-5 regarding diabetes also take the cut-off score of ≤ 50, reporting a sensitivity from 0.57 to 1.00 and specificity from 0.78 to 0.88 [20]. Other studies that examined the utility of the WHO-5 English Version as a screening instrument also took < 52 as a cut-off score, which displayed adequate sensitivity and specificity [46, 47]. We think the differences are mainly due to two reasons. The first is the index of validity. The most frequently chosen index of validities is CES-D ≥ 16, or PHQ-9 ≥ 10 in diabetes patients [46–49]. However, we used M.I.N.I. DSM-IV depression as a golden standard in diagnosis, which may lead to a discrepancy in the cut-off value. Compared with other studies using M.I.N.I. as a golden standard, our study showed comparable sensitivity and specificity at a lower cut-off value [33, 50]. Meanwhile, we conducted ROC analysis using the PHQ-9 as index of validity. The cut-off value 54 was also consistent with former research [41]. The second is the study population since our research was conducted specifically on outpatients with type 2 diabetes in general hospitals across China. The psychosocial factors including the conception of illness, socioeconomic conditions, and awareness of mental symptoms may account for the difference in the cut-off score [29].

The advantages of this study are as follows: First of all, this is the first study to examine the psychometric properties of WHO-5 in Chinese diabetic patients. Secondly, we used M.I.N.I. as the gold standard in determining the screening properties of WHO-5-C. M.I.N.I. is a structured diagnostic interview based on DSM-4 and ICD-10, which is more reliable for measuring depression than depression self-rating scales. Naturally, the study also has some limitations. First, the study was conducted in a third-class general hospital in China located in Beijing, and this may limit the generalization of the results across China, especially in primary care centers in rural or remote regions. Second, the participants were recruited between 2014 to 2015, the medication circumstances of which may be slightly different from the present. Thirdly, outcomes regarding the correlation between WHO-5-C and diabetes management self-efficacy and diabetes self-care behaviors are insufficient. The predictive validity of WHO-5-C should also be further investigated by reexamining the correlation with diabetic laboratory indicators, complications, mortality, and morbidity rate with a longitudinal study design.

## Conclusion

The WHO-5-C presents satisfactory reliability and validity among diabetes mellitus patients in China and can be considered a reliable screening tool for depression in diabetes patients. The recommended cut-off value of this study is 42.

## Data Availability

The datasets analyzed in this article are not publicly available. Requests to access the datasets should be directed to XH, hongxia@pumch.cn.

## Abbreviations

WHO-5: WHO-5 well-being index
WHO-5-C: Chinese version of WHO-5 well-being index
PHQ-9: Patient Health Questionnaire-9
PAID-20: 20-Item problem areas in diabetes
M.I.N.I.: Mini International Neuropsychiatric Interview
HAM-D: Hamilton Rating Scale for Depression
CFA: Confirmatory factor analysis
INTERPRET-DD: International Prevalence and Treatment of Diabetes and Depression Study
SPSS: Statistical Package Social Sciences
CFI: Comparative Fit Index
TLI: Tucker-Lewis Index
RMSEA: Root Mean Square Error of Approximation
ROC: Receiver Operating Characteristic
KMO: Kaiser–Meyer–Olkin measure of sampling adequacy
AUC: Area Under the Curve
BMI: Body Mass Index
HbA_1c_: Glycated Hemoglobin A1c
LDL-C: Low-Density Lipoprotein Cholesterol
HDL-C: High-Density Lipoprotein Cholesterol
TG: Triglycerides
TC: Total Cholesterol
